# Applying innovative technological interventions in the preservation and packaging of fresh seafood products to minimize spoilage - A systematic review and meta-analysis

**DOI:** 10.1016/j.heliyon.2024.e29066

**Published:** 2024-04-04

**Authors:** Shahida Anusha Siddiqui, Shubhra Singh, Nur Alim Bahmid, Abhilash Sasidharan

**Affiliations:** a Technical University of Munich, Campus Straubing for Biotechnology and Sustainability, Essigberg 3, 94315 Straubing, Germany; b German Institute of Food Technologies (DIL e.V.), Prof.-von-Klitzing Str. 7, 49610, Quakenbrück, Germany; c Department of Tropical Agriculture and International cooperation, National Pingtung University of Science and Technology, 91201, Taiwan; d Research Center for Food Technology and Processing, National Research and Innovation Agency (BRIN), Gading, Playen, Gunungkidul, 55861, Yogyakarta, Indonesia; e Department of Fish Processing Technology, Kerala University of Fisheries and Ocean Studies, Panangad P.O 682506, Kerala, India

**Keywords:** Seafood, Preservation, Packaging, Spoilage

## Abstract

Seafood, being highly perishable, faces rapid deterioration in freshness, posing spoilage risks and potential health concerns without proper preservation. To combat this, various innovative preservation and packaging technologies have emerged. This review delves into these cutting-edge interventions designed to minimize spoilage and effectively prolong the shelf life of fresh seafood products. Techniques like High-Pressure Processing (HPP), Modified Atmosphere Packaging (MAP), bio-preservation, and active and vacuum packaging have demonstrated the capability to extend the shelf life of seafood products by up to 50%. However, the efficacy of these technologies relies on factors such as the specific type of seafood product and the storage temperature. Hence, careful consideration of these factors is essential in choosing an appropriate preservation and packaging technology.

## Highlights

• Seafood spoilage is the deterioration of seafood products as a result of microbial, enzymatic, or oxidative changes.
• The prevention of seafood spoilage is important to guarantee food safety.
• Cold chain is vital for preserving seafood quality by sustaining the ideal low temperatures throughout the process.
• Thermal and non-thermal technologies inhibits seafood spoilage ensuring extended shelf life.
• The smart packaging of seafood enables us to monitor and control spoilage aspects during storage and transportation.

## Graphical abstract

**Figure:**
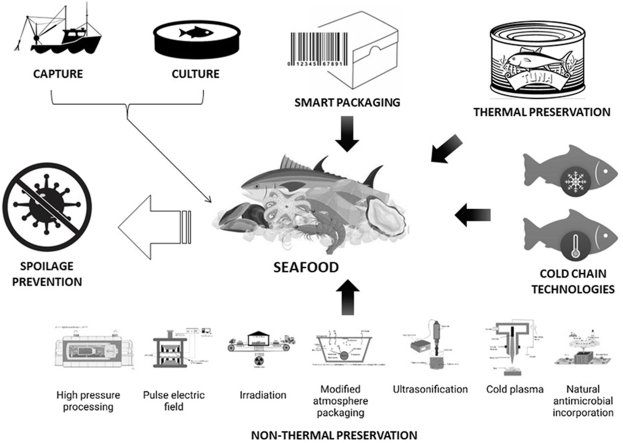

## 1 Introduction

Seafood stands as a valuable nutritional resource, offering crucial elements like protein, omega-3 fatty acids, vitamins, and minerals, with a particular emphasis on its provision of lean proteins. According to reports from the Food and Agriculture Organization (FAO), seafood contributes to 6 percent of dietary protein globally [1,2]. Notably, fish lipids boast high levels of omega-3 fatty acids, reducing the risk of myocardial infarction and delivering various other health benefits. Embracing seafood as part of a balanced diet is essential in mitigating the risk of numerous chronic diseases [3]. Additionally, seafood is a rich source of iodine, crucial for thyroid function, with a recommended daily intake of 150 μg/d for adults [4]. Beyond its nutritional significance, seafood plays a pivotal role in global nutritional and economic security, serving as a vital source of sustenance and income for millions, particularly in developing nations. In numerous coastal communities, seafood constitutes a substantial portion of daily protein intake, and the livelihoods of millions hinge on the fishing industry [5].

Current trends in production highlight a global increase in seafood consumption, with a significant emphasis on sustainability. Despite this positive direction, challenges such as overfishing, habitat destruction, pollution, and climate change pose substantial threats to sustainability [6]. Consequently, there is a growing recognition of the need for sustainable seafood production practices to ensure the long-term viability of the fishing industry and promote ocean health. Another emerging concern is the impact of the seafood industry on global warming, prompting studies that assess and compare greenhouse gas emissions from seafood and other food products, providing insights into emissions per kilogram of product [7]. Many countries, recognizing the nutritional benefits, recommend seafood consumption as a key component of a healthy diet [8]. The global nature of seafood as a highly traded commodity is influenced by various factors. Cultural preferences in many countries prioritize seafood, whether wild-caught or farmed [[9], [10], [11]]. Furthermore, the processing of most seafood products occurs in one location before being transported elsewhere for consumption, involving necessary preservation methods such as freezing or canning [12].

Global fish waste generation is a significant global concern, with estimates suggesting that between 27% and 39% of the total annual fish catch is lost through processing side streams [12]. Post-harvest losses, particularly prevalent in developing nations, impact a substantial portion of farmers, with around 35% of the total harvest in fisheries experiencing losses [13,14]. Improper handling, storage, preservation, and packaging of seafood contribute to rapid quality deterioration, leading to spoilage and nutrient loss. Spoiled seafood not only presents unpleasant taste and odour but also poses potential health risks to consumers. In many developing countries lacking adequate handling, storage, and preservation practices, nutritional loss exacerbates the challenge, adversely affecting food security by diminishing the availability of this vital protein source and reducing income for stakeholders.

Moreover, the economic repercussions of post-harvest loss extend to fishermen, seafood businesses, and the broader economy. In the case of inland capture and aquaculture species, adopting contemporary harvesting technology and increasing storage capacity at landing and selling locations is recommended [15]. Studies in Bangladesh highlight significant income losses among fish farmers and markets due to post-harvest fish loss. Factors contributing to such losses include poor packaging and handling practices, improper collection, inadequate transportation, and a lack of infrastructure facilities. Creating awareness of these challenges and implementing improvements in infrastructure and transportation facilities can play a pivotal role in reducing post-harvest losses [16].

Advanced preservation techniques play a pivotal role in thwarting the spoilage of seafood and prolonging its shelf life. Recent advancements include the proposal of dietary biomarkers and related assessments for more precise measurement of seafood intake, facilitating accurate studies on consumption rates [17]. These techniques contribute significantly to upholding seafood quality and safety, thereby mitigating the risk of post-harvest loss due to spoilage and enhancing the availability of this crucial nutritional source [18]. One such technique is Modified Atmosphere Packaging (MAP), involving the adjustment of oxygen, carbon dioxide, and nitrogen levels within the packaging to reduce the growth of microorganisms and delay spoilage [19]. High-Pressure Processing (HPP) applies high pressure to seafood products, eliminating bacteria, viruses, and harmful microorganisms, thereby extending shelf life by impeding the growth of spoilage organisms. Irradiation utilizes ionizing radiation to eliminate harmful microorganisms, also reducing the growth of spoilage organisms and extending the shelf life of seafood [20].

Vacuum packaging, another method, removes air from the packaging, diminishing the growth of aerobic spoilage organisms and preserving seafood freshness for a more extended period. Additionally, active packaging involves materials releasing antimicrobial agents, antioxidants, or other substances to delay spoilage, further enhancing the quality and safety of seafood fresh produce [21,22]. By extending the shelf life of seafood, these techniques contribute to waste reduction, increased profitability for seafood businesses, and overall food security. In this comprehensive review, we present detailed information on various preservation technologies adopted to maintain and extend the shelf life of seafood products. The discussion covers mechanisms of seafood spoilage, indicators, detection technologies, and emerging non-thermal preservation methods, providing a holistic understanding of the subject.

## 2 Search strategy and selection criteria for meta-analysis

**Fig. 1:**
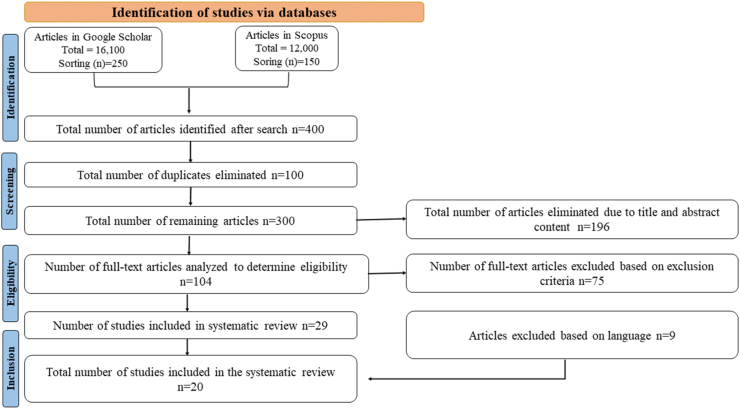
Preferred reporting items for systematic reviews and meta-analysis (PRISMA) flow diagram.

The present review was reported in accordance with 2009 PRISMA statement. For that we have conducted a scoping review to identify areas of research consistently held to provide for the new interventions in seafood preservation and packaging (Fig. 1). Studies were focussed on six areas (1) different seafood spoilage and how to detect them (2) innovative technological interventions (3) pre/post-harvest transportation and storage, (4) low temperature technologies used for preservation, (5) high-temperature technologies used for storage and preservation, and (6) non-thermal technologies.

We searched for articles and reviews in these six areas using Google scholar, Scopus and PubMed. Searches were conducted from 2010-1023 using terms ("Seafood preservation" AND "packaging" OR "spoilage") AND (“innovative” OR “technological interventions”) and limited searches to the title and abstract to exclude irrelevant hits. Inclusion criteria were designed to identify the best available evident in each research area and consisted of.

i. Studies published as peer-reviewed literature, narrative reviews and systematic reviews
ii. Studies with randomised controlled trials, quasi-experimental studies and observational studies that assess the impact of innovative technological interventions on the preservation and packaging of fresh seafood.
iii. Studies with clear and relevant outcome measures such as spoilage reduction, shelf-life extension and maintenance of product quality
iv. Studies that are directly applicable to human consumption rather than solely focussed on laboratory or animal experiments
v. Studies that are accessible to full text for comprehensive evaluation of the methodology and results

Exclusion criteria consisted of.

i. Studies with non-English language
ii. Studies published between 2010 and 2023
iii. Case reports, dissertations, or studies with insufficient methodological details
iv. Studies reporting the same data to avoid redundancy
v. Studies that focus on the preservations and packaging of products other than fresh seafood
vi. Studies with small sample size that lack statistical power
vii. Studies that include terrestrial animals

## 3 Seafood spoilage and detection mechanisms

Fresh seafood is highly perished due to its characteristics of being short shelf-life therefore it must be stored properly to avoid deterioration and ensure microbial safety and marketable shelf-life especially with the seafood. Consumers mostly prefer fresh and less processed foods since the health-related concerns regarding preservatives bothers them. The main problem is seafood spoilage as sometimes storage procedures and food packaging systems are compromised. Seafood spoilage refers to the deterioration of the quality, flavour, texture, and nutritional value of seafood because of microbial, enzymatic, and chemical reactions that occur during storage and handling. Spoilage can be caused by a variety of factors, including exposure to oxygen, temperature abuse, poor sanitation, and physical damage. Spoiled seafood may have an unpleasant odour, slimy texture, discoloration, and off-flavours. In addition to affecting the quality and taste of seafood, spoilage can also pose a health risk to consumers by increasing the risk of foodborne illness. Proper handling, storage, and cooking can help prevent seafood spoilage and ensure food safety [23].

**Fig. 2:**
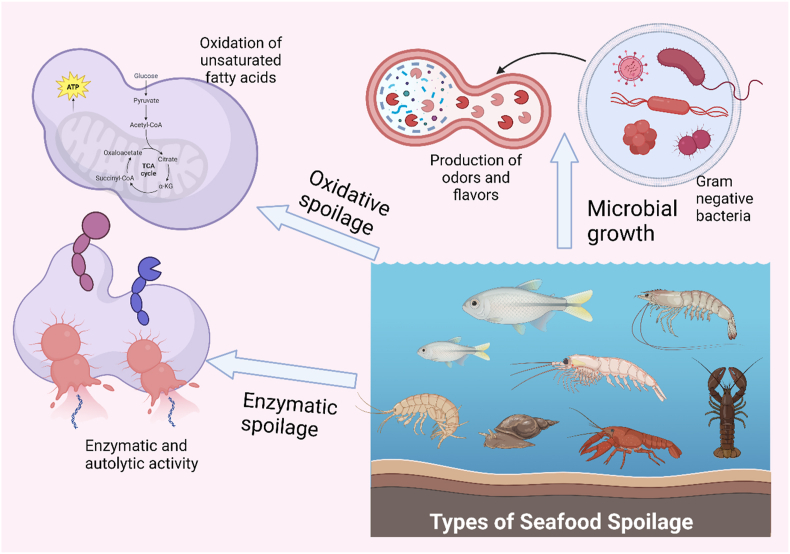
Illustration of major types of seafood spoilage (Created with Biorender.com).

**Fig. 3:**
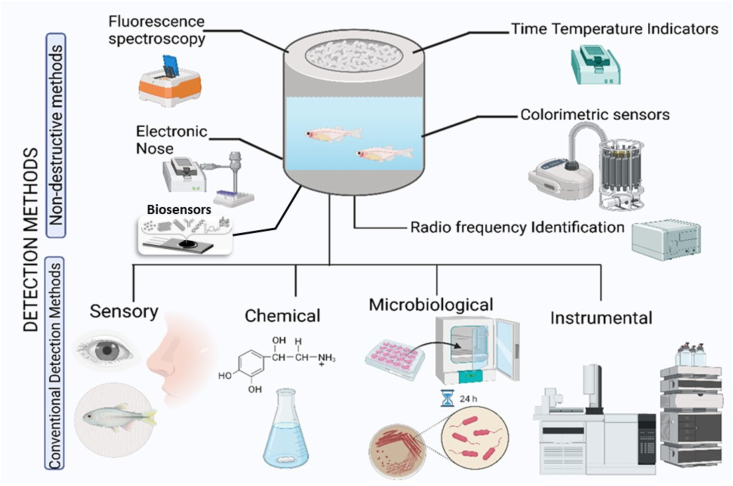
Illustration of different types of detection methods for spoilage in seafood (Created with Biorender.com).

Seafood spoilage can be classified into several types based on the cause and characteristics of the spoilage. Fish spoilage have three basic mechanisms: enzymatic, microbial growth and chemical spoilage as shown in Fig. 2 and Table 1 [24]. Enzymatic spoilage occurs when enzymes present in the seafood break down its proteins, lipids, and carbohydrates, causing texture changes, off-flavours, and discoloration. The two main types of enzymes involved in enzymatic spoilage with autolytic enzymes in seafood are proteases and lipases. Proteases break down proteins into amino acids, while lipases break down fats into fatty acids and glycerol. These enzymes are present in high levels in certain types of seafood, such as tuna, mackerel, and herring. Microbial spoilage is caused by the growth of bacteria, yeast, and moulds on the seafood, which can lead to off-odours, slime formation, and discoloration [[23], [24]]. Chemical spoilage happens when chemical reactions take place in the seafood due to exposure to oxygen, light, or other factors, resulting in off-flavours, discoloration, and rancidity. Physical spoilage occurs when seafood is physically damaged, such as through rough handling or freezing, leading to changes in texture and flavour [25]. Proper handling, storage, and cooking can help prevent seafood spoilage and ensure food safety. However, with the advent of novel molecular techniques, the study of seafood characteristics during processing, storage and distribution can be assessed with the multi-omics approaches. This approach specifically marks for the functionalities in the complex food matrix including the unexpected spoilage situations. Such as some of the instrumental techniques available for detecting the extent of each type of seafood spoilage (Fig. 3) [26].

**Table 1:** Mechanisms of fish spoilage.

I. Autolytic spoilage						References
	Enzyme(s)	Substrate	Effect	Detection methodology	Limitations	
1	Glycolytic enzymes	Fish or shellfish shell	Lactic acid production resulting in pH drop	Texture, odours and flavours	pH, temperature, and storage conditions	[19]
2	Proteases	Fish or shellfish shell	Soft, slimy, and discoloured	Caseinolytic activity	pH, temperature, and storage conditions	[27]
3	Lipases	Fish or shellfish shell		Titration assay	pH, temperature, and storage conditions	[96)
**II. Oxidative spoilage**						
	**Oxidation target compound**	**Effect**		**Detection methodology**	**Limitations**	
1	Free fatty acids, aldehydes, ketones and hydroperoxides	Rancid flavour and off-texture and odour		Gas chromatography or electronic noses, sensory analysis, colorimetric assays	processing and storage conditions	[28]
**III. Microbial spoilage**						
	**Specific spoilage bacteria**	**Spoilage compound**		**Detection methodology**	**Limitations**	
1	*Pseudomonas* spp.	trimethylamine (TMA)		Plate counts or molecular methods such as PCR, chromatography, and colorimetric methods for measuring compounds	Type, processing, and storage conditions of seafood	[29,30]
2	*Shewanella* spp.	dimethylamine (DMA)		Molecular methods, chromatographic and colorimetric methods	Low levels of compounds, survival of bacteria at low temperatures	[29,30]
3	*Photobacterium* spp.	ammonia		Molecular methods, chromatographic and colorimetric methods	Low levels of compounds, survival of bacteria at low temperatures	[29]

Microbial spoilage can be detected using microbiological techniques such as plate counts, PCR-based methods, and rapid detection kits. Plate counts involve culturing bacteria, yeast, and moulds from seafood samples on nutrient-rich agar plates, followed by counting and identification of the colonies. PCR-based methods use DNA amplification to detect and identify specific microbial species, while rapid detection kits use immunological or enzymatic reactions to detect microbial spoilage [31]. Enzymatic spoilage can be detected using enzymatic assays that measure the activity of enzymes such as proteases, lipases, and amylases in seafood samples. These assays can provide information on the degree of enzymatic degradation of the seafood [19]. Chemical spoilage can be detected using instrumental techniques such as gas chromatography-mass spectrometry (GC-MS), high-performance liquid chromatography (HPLC), and Fourier transform infrared spectroscopy (FTIR). These techniques can identify and quantify chemical compounds that are indicative of spoilage, such as volatile organic compounds (VOCs) and lipid oxidation products [32]. An innovative analytical approach as duality-by-design was used by researchers to optimize the experimental parameters of headspace solid-phase microextraction (HS-SPME) and GC-MS conditions for the quantification of volatile amines found as spoilage index in seafood products. The optimization results in the selection of the best operable design for the acceptable quality of seafood [33]. Physical spoilage can be detected using visual inspection and texture analysis techniques. Visual inspection involves assessing the appearance and colour of seafood samples, while texture analysis involves measuring the firmness, elasticity, and cohesiveness of the samples using instruments such as texture analysers. Such as in a study, simple and cost-effective filter papers loaded with aggregation-induced emission luminogen to detect salmon spoilage. The quantification is done based on the intensity of the luminogen which increases with increase in spoilage, and it can be used for on-site to evaluate the quality of seafood spoilage [34].

Non-destructive sensor-based rapid seafood spoilage detection methods have gained popularity in recent years due to their fast and non-invasive nature. Electronic nose (E-nose) is a commonly used sensor array that can detect and recognize volatile organic compounds (VOCs) released by seafood during spoilage. This technology can provide a rapid indication of the extent and type of seafood spoilage. Electronic tongue (E-tongue) is another sensor array that can detect and recognize taste and flavour changes in seafood samples. E-tongue technology can provide a rapid indication of the degree of spoilage in seafood products. In (NIR) is another non-destructive technique that can detect changes in the molecular structure of seafood during spoilage. This technique can provide rapid and accurate information on the degree of spoilage in seafood samples. Biosensors with enzyme, Aptamer, cellular, antibody or DNA based receptors interacting with electrochemical, optical, or mass detection technology based transducers combined with signal amplification and detection mechanisms are another group of spoilage detection mechanism which finds application in seafood processing [35]. Overall, non-destructive sensor-based rapid seafood spoilage detection methods can offer a reliable and efficient means of detecting seafood spoilage, helping to ensure food safety and quality [36].

Fish spoilage, marked by off-odours such as sweet, fruity, ammonia-like, putrid, and sulfuric, is closely linked to the release of volatile organic compounds. Effectively characterizing the profile of these compounds is crucial for controlling spoilage and ensuring the efficient transportation of seafood under refrigerated conditions. To achieve this, it is imperative to establish suitable quantification methods. Multivariate statistical analysis stands out as a potent tool for identifying potential indicators of seafood spoilage. One widely employed technique in multivariate statistical analysis is Principal Component Analysis (PCA). PCA involves transforming data into a set of new variables that are uncorrelated and ordered by the variance they explain in the original dataset. This enables researchers to identify key variables strongly associated with seafood spoilage, unveiling patterns and relationships in large datasets that may have been previously overlooked. Discriminant Analysis is another valuable multivariate statistical technique for pinpointing potential indicators of seafood spoilage. This method seeks the linear combination of variables that best separates groups of samples based on their spoilage status. Identifying which variables are most effective in predicting spoilage or non-spoilage helps develop models for predicting spoilage based on measurable indicators. By delving into large datasets and uncovering patterns and relationships between variables, researchers gain valuable insights into the factors contributing to seafood spoilage. This, in turn, aids in the development of more accurate models for predicting spoilage based on measurable indicators [37]. The limits for permissible extent of spoilage vary according to different food standards and regulations.

Here are some examples of permissible limits of seafood spoilage as per different food standards [25].

a. European Union (EU): The EU has set maximum acceptable limits for histamine, which is a toxic compound that can be produced during the spoilage of certain types of seafood. The limit is 200 mg/kg for fish products and 400 mg/kg for fishery products. The EU also has microbiological criteria for seafood, which include limits for specific bacterial species and total viable counts.
b. United States Food and Drug Administration (US FDA): The US FDA has set maximum limits for certain chemical and physical parameters that indicate seafood spoilage, such as histamine, trimethylamine oxide (TMAO), and total volatile basic nitrogen (TVBN). The limits vary depending on the type of seafood and range from 20 to 60 mg/100g for histamine, 600–900 mg/100g for TMAO, and 30–60 mg/100g for TVBN.
c. Codex Alimentarius: The Codex Alimentarius, a joint international food standards organization of the World Health Organization (WHO) and the Food and Agriculture Organization (FAO), has established microbiological criteria for seafood products. The criteria include limits for specific bacterial species and total viable counts.
d. Japan: The Japan Ministry of Health, Labour, and Welfare has established limits for histamine, TMAO, and TVBN, similar to those of the US FDA. Japan also has specific limits for total aerobic plate counts, coliforms, and E. coli in seafood.

## 4 Significance of pre/post-harvest handling, transportation and storage on seafood spoilage

The microbiota of fish and fish products are highly affected by environmental factors such as spoilage microorganisms that are often found in water streams and sediments during pre-harvest conditions. The pre-harvest conditions vary according to the different regions in which fishes are inhabited and one of the important source is water from sea, lakes, rivers, or wells. The quality of water determines the growth and development of aquatic species from fishes to crustaceous. The physiochemical property of water is affected by the seasons and climatic conditions such as occurrence of species like *Aeromonas hydrophila* and *Psuedomonas fluorescens* have positive correlation with dissolved oxygen levels and maintaining the temperatures. However, the seasonal change affect the population of these species in the water bodies [38]. Climatic conditions such as rainfall contribute to bacterial contamination of surface water. In comparison with water, sediments consist of higher diversities of bacterial communities along with gut microbiome of fish found in culture practices and other wild locations. Therefore, occurrence of contaminants in sediments is influenced by the collecting timings and locations of fishes. Some of the researchers have indicated that the open systems in aquaculture practices where both uneaten fish feed and fish excrement transfers freely poses high risks of contamination to the surrounding environment. These practices have adverse effect on the diversity and abundance of the microbial contaminant in the sediment during pre-harvesting. Other factors include intrinsic factors for example genetics and extrinsic factors such as water sources and regular aquaculture practices [29].

Other seafood such as is warm water penaeid (saltwater) shrimps that are grown in earthen ponds 4–6 feet deep. Before consumption, shrimp has to undergo HACCP Critical Control Point, however certain pathogen (*Salmonella*) is found in most of the warm-blooded animals and mostly present in the intestinal tract of farmed shrimps. The present of this pathogen is an indication of poor sanitation during production and harvesting according to U.S. Food and Drug Administration (FDA). Usually, shrimp price is affected by the evaluation of conditions based on shell texture, flavour and odour. Shrimps are often microwaved to check the off-odours and off-flavours and off-colours in the red head is detected by visual defects. Rapid deterioration is observed after harvesting therefore the quality of shrimp is primarily maintained at low temperatures such as in ice waters. Post harvesting procedures include dipping the shrimps in solutions before storing in ice for transport to the processing plant and chemicals are used to stabilise the properties of shrimp such as 100 ppm sodium metabisulfite and 50 ppm 4-hexylresorcinol with ice water [39]. Similarly, Bivalve molluscan shellfish, such as clams, oysters, and mussels, are a valuable source of protein and nutrients for people around the world. However, to ensure their safety and quality, it is important to follow proper pre-harvest and post-harvest processing techniques. Pre-harvest processing involves selecting a suitable site for farming the shellfish, monitoring water quality, seeding the site with young shellfish, and timing the harvest to coincide with maturity (Table 2). Post-harvest processing includes depuration to purge contaminants, grading and sorting by size and quality, packaging, and storage in a cool, clean environment, and finally cooking and preparation according to the consumer's preference. By following these processing techniques, the risk of contamination can be minimized, and the flavour and texture of the shellfish can be maintained, providing consumers with a delicious and nutritious food source [1,39].

**Table 2:** Adaptive approaches in pre-and post-harvest losses of seafoods.

Pre-harvest Approaches			
Approach	Description	Examples	References
Improved fishing practices	The use of more efficient and sustainable fishing practices to reduce bycatch and minimize damage to non-target species.	Using fishing gear that is designed to target specific species while avoiding non-target species. The use of escape devices, such as turtle excluder devices in shrimp trawling, to allow non-target species to escape from fishing gear.	[10]
Sustainable aquaculture practices	To improve the sustainability of seafood production and reduce losses	Using sustainable feeds, such as algae or insects, to reduce waste and environmental impacts. Implementing best management practices, such as minimizing disease outbreaks and reducing pollution, to improve the sustainability of aquaculture operations.	[40]
Early detection and monitoring	Early detection and monitoring of potential issues, such as disease outbreaks or environmental changes, can help to prevent losses.	Using remote sensing technologies to monitor environmental conditions, such as water quality or temperature. Conducting regular health checks on farmed or harvested seafood to detect disease early.	[41]
**Post-harvest Approaches**			
Improved handling practices	Improved handling during processing and storage to reduce spoilage and maintain product quality.	Using ice or refrigeration to keep fish cool and prevent spoilage. Minimizing the time between harvest and processing to maintain freshness.	[42]
Value-added products	Processing seafood into value-added products, such as fillets or ready-to-cook products, can increase the value and shelf life of the product.	Processing fish into fillets or portions to make them more convenient for consumers. Developing new products, such as fish burgers or fish nuggets, to appeal to different consumer preferences.	[43]
Cold chain management	The use of appropriate refrigeration and cold chain management practices can minimize spoilage and extend the shelf life of seafood.	Using temperature monitoring systems to ensure that seafood remains within the appropriate temperature range.	[42]
Modified atmosphere packaging	To maintain product quality and extend the shelf life of seafood by controlling the gas composition in the packaging.	Vacuum packaging to remove oxygen and prevent spoilage. The use of gas flushing to create a modified atmosphere that inhibits the growth of spoilage organisms.	(47)
Emerging preservation technologies	It can be used to reduce spoilage and extend the shelf life of seafood.	High-pressure processing to inactivate spoilage organisms and enzymes. Pulsed electric field processing to disrupt cell membranes and inhibit spoilage organisms.	[20]

**Fig. 4:**
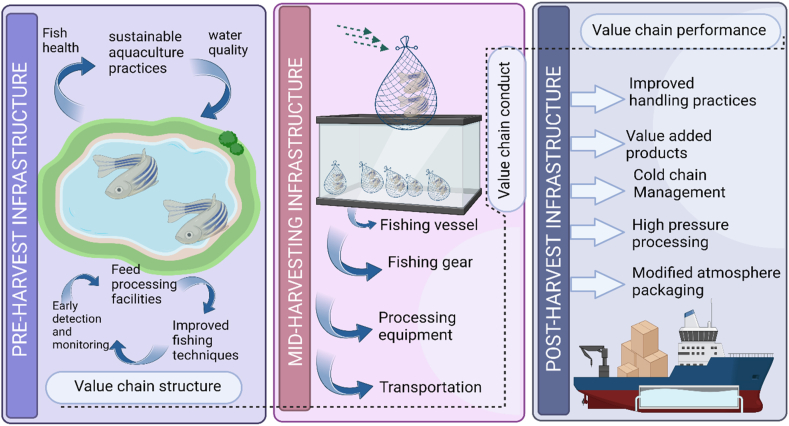
Value chain of pre/post-harvest seafood handling infrastructure (Created with Biorender.com).

According to Ref. [12], fish loss has been estimated to be 37% as the total fish caught each year and post-harvest losses remains a global issue in mostly in the low-income countries where population is dependent on the production of fish for food, nutrition, and income [13,44]. Post-harvest losses are measured as the reduction in the quality and quantity of the fish produced at different stages in the value chain (Fig. 4). For example, physical loss, when the fishes are not handled properly and are completely lost in the value chain. Another one is quality of fish which is harmed by the fluctuating monetary value. Market also plays an important role and creates a production cost or gluts that causes process of seafood (mostly fishes) to drop. More loss is associated with nutritional value which is affected by biochemical changes that occurs due to spoilage, processing, meal preparation etc. However, out of all these post-harvest losses, quality is the most important and common type as it accounts for 70% of total loss. These losses are accountable during processing, transport, marketing techniques [14,45]. For instance, in a country like Zambia, where most of the population is dependent on fishery, people in this business uses drying racks to place their fish that are easily exposed to the environmental infections and creatures. Similarly, in Barotse floodplain, sack like canoes are used to transport fish to the local market but the quality of fish is compromised due to lack of proper transportation. For long distance transportation, fishes are often stacked over one another in buses or trucks that might get broken over the distance reducing the quality and value of fishes. For the people working in the value chain, as the quality of seafood or fish degrades because of poor handling or transportation, the market price is reduced hence affecting the income of fish handlers [[15], [24]].

Therefore, there is need to develop low-cost processing technologies for fish drying techniques that can tackle quality issues without pushing the prices. Such as WorldFish, an IRDC funded project in Zambia where local communities are practicing adaptive learning approaches. This approach acknowledges uncertainties and complexity of socio-ecological systems, providing a well-structured approach for learning and adapting adjustments in fisheries [46]. There are other methods that are designed by Trade and Marketing Service of the Fisheries and Aquaculture Department (formerly the Fish Utilization and Marketing Service) in FAO for addressing a regional post-harvest loss assessment (PHLA) programme. This programme lasted for 18 months in started from October 2006, where fishery officers were provided with qualitative and quantitative fish loss assessments, capacity building techniques with planned support and supervision. The loss assessments were studied in five Sub-Saharan African countries (Ghana, Kenya, Mali, United Republic of Tanzania, and Uganda). Various coping strategies were suggested to the fisherman, processors, and traders of these regions however careful and continued technical and policy initiatives are needed by International and national organizations. Additionally, governments and development agencies must ensure fisheries-related policies and practices for loss assessment in their region to create experience and nullify the losses [24].

Recently, many researchers have reported the influence of stress conditions on fish physiology in aquaculture industry that can affect pre- and post-harvest losses. Aquaculturists can manage the stress related conditions such as stocking density, oxygen level, water density, exposure to disease, quality of feed, handling, and breeding practices. Reducing the pre and post harvesting losses means to decrease stress conditions in fishes as stress leads to physiological responses with physical changes of increased heart rate and muscle contractions which compromises the quality of the fish transported. As reported, fish under stress during harvesting go into rigorous death exhibiting drip loss and softening the muscle texture. In addition, stress conditions in pre and post harvesting can induce peroxidase and aldehyde production which decreases the nutritional quality of the fish fillet leading to failure in consumer acceptance. Therefore, utilising catch methods should be considered that could decrease fish stress during harvesting procedure. Other methods during post-harvesting must be applied including asphyxiation on ice, bleeding when fishes are conscious, percussive stunning, spiking and electrical stunning. Moreover, the best method should have least impact on the quality of fishes [47].

Onboard seafood handling and cold chain infrastructure facilities have significantly evolved in recent years to prevent seafood spoilage and ensure that consumers receive fresh and safe products. Here are some of the innovative methods that are being used to prevent different seafood spoilage conditions [42].

i. Temperature Monitoring and Control: One of the most critical aspects of preventing seafood spoilage is maintaining the right temperature. Modern onboard seafood handling and cold chain facilities use advanced temperature sensors and control systems to monitor and adjust the temperature at various stages of the supply chain. This helps to prevent spoilage caused by temperature fluctuations [13].
ii. Modified Atmosphere Packaging (MAP): Another effective technique for preventing seafood spoilage is Modified Atmosphere Packaging (MAP). This technique involves modifying the composition of the air in the packaging to slow down the growth of bacteria and other microorganisms. MAP is particularly effective for preventing spoilage caused by aerobic bacteria [21].
iii. Rapid Freezing: Rapid freezing is another technique used to prevent seafood spoilage. This technique involves freezing the seafood at very low temperatures (−40 °C to −60 °C) within a short period (usually within hours of catching). Rapid freezing helps to preserve the quality and freshness of the seafood and prevents the growth of bacteria and other microorganisms [42].
iv. High-Pressure Processing (HPP): High-Pressure Processing (HPP) is another method used in onboard seafood handling and cold chain facilities. This technique involves applying high pressure (up to 600 MPa) to the seafood to inactivate microorganisms and enzymes that cause spoilage. HPP is particularly effective for preventing spoilage caused by psychotropic bacteria [20].
v. Vacuum Packaging: Vacuum packaging is another technique used to prevent seafood spoilage. This technique involves removing air from the packaging, creating a vacuum that inhibits the growth of bacteria and other microorganisms. Vacuum packaging is particularly effective for preventing spoilage caused by anaerobic bacteria [48].

Non-destructive evaluation (NDE) technologies are increasingly becoming an essential part of the cold chain logistics of seafood. NDE technologies use non-invasive methods to detect and evaluate defects, and changes in material properties without causing damage to the product. In addition to that, they can offer accurate physical and chemical information of seafood such as protein, water content, fat, and other biological properties [49]. Preliminary studies have indicated that monitoring technologies such as gas-sensitive freshness indicator and Radio Frequency Identification (RFID), can effectively monitor changes in seafood quality and record the cold chain conditions [41]. Non-destructive detection such as machine vision systems (MVS), nuclear magnetic resonance (NMR), spectroscopic techniques and electric nose establishes rigorous verification systems to ensure high throughput monitoring results [50]. There are several other monitoring available in the seafood industry, including magnetic resonance imaging (MRI), X-ray imaging, ultrasonic testing (UT), and infrared thermography (IRT). MRI is used to create detailed images of seafood products, allowing for the detection of defects and changes in internal structures. X-ray imaging is used to detect bone fragments and other contaminants in seafood products. UT uses sound waves to detect internal defects in seafood products, while IRT is used to measure the temperature of seafood products and identify potential hotspots that can cause spoilage [51].

Research trends in NDE technologies in the seafood industry have focused on the development of new methods for the detection and evaluation of seafood products. One area of research has been the use of spectroscopy to detect and quantify contaminants and nutrients in seafood products. Acoustic emission testing is another area of research, which uses sound waves to detect and locate defects in seafood products. Laser-induced breakdown spectroscopy (LIBS) is a promising technology that uses lasers to vaporize small amounts of seafood product, which are then analysed to detect and quantify contaminants. With ongoing research and development, it is likely that new NDE technologies will continue to emerge in the seafood industry, offering even greater benefits for the monitoring and maintenance of seafood products throughout the supply chain [52].

Blockchain technology has emerged as a promising solution to enhance traceability, transparency, and accountability within the seafood industry, addressing various issues related to supply chain management and sustainability. A key application of blockchain in the seafood sector is traceability, allowing for the tracking of seafood products from the point of catch to the point of consumption. This technology facilitates the identification of the product's origin, the conditions of its capture, and the subsequent handling and processing it underwent. Despite its potential benefits, the adoption of blockchain in the seafood industry faces challenges, with one of the primary limitations being the high cost associated with implementation and maintenance. The considerable investment required in infrastructure, software, and personnel can be a deterrent for small and medium-sized enterprises within the seafood sector. However, the advantages of blockchain technology in the seafood industry cannot be overlooked. One notable benefit is the increased transparency and accountability it provides. Blockchain offers a secure and transparent record of transactions across the supply chain, making it easier to detect and address issues like fraud, mislabeling, and illegal fishing. Moreover, blockchain aids in improving food safety by enabling real-time tracking of products and identifying potential sources of contamination. In conclusion, while challenges such as cost may hinder the widespread adoption of blockchain technology in the seafood industry, its potential applications and the benefits it offers make it an attractive solution for enhancing traceability and sustainability in the seafood supply chain. As the technology evolves and becomes more cost-effective, it is anticipated that more companies in the seafood industry will embrace blockchain to enhance their operations and meet consumer demands for transparency and accountability [40].

## 5 Low temperature technologies for spoilage prevention

Low-temperature technologies are commonly used to prevent spoilage in seafood. These technologies include the use of ice, chilling, super-chilling, freezing, different types of freezers, freezing mediums, freeze drying, low-temperature packaging, and the use of additives (Table 3). Some of the low-temperature techniques are explained below [53,54].

**Fig. 5:**
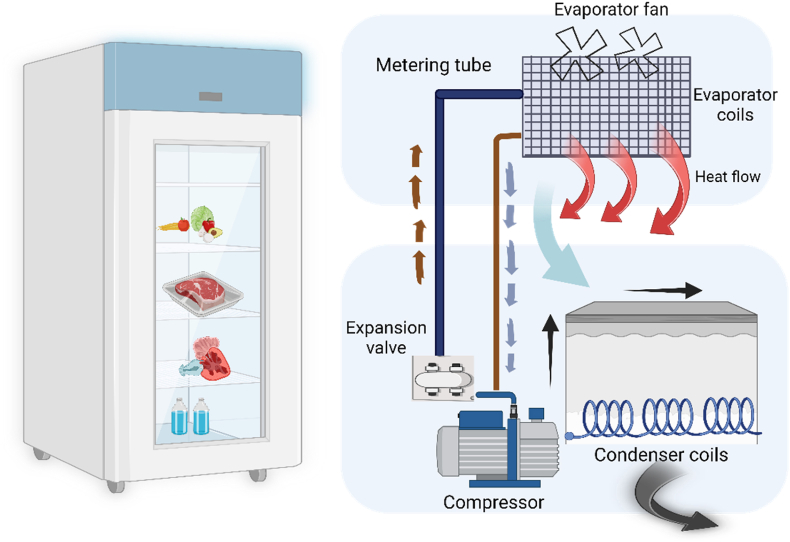
Illustration of basic freezing principle with instrumental components (Created with Biorender.com).

1. Ice: Ice is commonly used to maintain the low temperature of seafood and prevent spoilage. Slurry ice and contact icing are the two primary methods used for this purpose.
2. Chilling: Chilling involves lowering the temperature of seafood to 0–5 °C to slow down the growth of bacteria and enzymatic activity. This method is commonly used for short-term storage of seafood. For example, fish is perishable food and chilling and freezing are the most common preservation techniques used for safe consumption. To increase the shelf-life of the fish species, whole, gutted or fillet are stored in chilling temperatures for transportation and processing over 14 days of time [55].
3. Super-chilling: Super-chilling involves lowering the temperature of seafood to −1 °C or below to extend its shelf life. This method is commonly used for fresh seafood transportation and storage.
4. Freezing: Freezing involves lowering the temperature of seafood below −18 °C to stop bacterial growth and enzymatic activity. This method is commonly used for long-term storage of seafood (Fig. 5).
5. Types of freezers: Different types of freezers used for seafood freezing include air blast freezers, plate freezers, and cryogenic freezers.
6. Freezing mediums: Common freezing mediums used for seafood freezing include brine, Carbon dioxide, and liquid nitrogen.
7. Freeze drying: Freeze drying is a method that involves removing moisture from seafood while maintaining its nutritional value and flavour.
8. Low-temperature packaging: Low-temperature packaging involves the use of packaging materials that can withstand low temperatures and prevent freezer burn. For example, low temperature of fish during harvest and transport improves quality and prolongs shelf life of Rohu in Myanmar ensuring quality and longer shelf life, as well as preventing contamination [56].
9. Additives used: Additives such as salt, vinegar, and citric acid are commonly used to preserve seafood by inhibiting bacterial growth.
10. Storage conditions: Proper storage conditions, such as temperature control, humidity control, and air circulation, are essential to prevent spoilage in seafood.

**Table 3:** Low temperature preservation technologies for seafood.

No	Preservation temperature (Target core temperature)	Type of technology	Additives used	Packaging material used	Storage conditions	Preferred shelf life	References
1	0–5 °C	Chilling	none	Vacuum packaging, modified atmosphere packaging	Maintain temperature, avoid exposure to air and moisture	2–3 days for fresh, avoid exposure to air and moisture	[21]
2	Below 0 °C	Super-chilling	none	Vacuum packaging, modified atmosphere packaging	Maintain temperature, avoid exposure to air and moisture	2–3 weeks for fresh seafood, up to 1 month for processed seafood	[42]
3	Below −18 °C	Freezing	none	Vacuum packaging, modified atmosphere packaging or freezer-safe containers	Maintain temperature, avoid exposure to air and moisture	6–12 months for most seafood, up to 18 months for certain types of seafood	[57]
4	Below −50 °C	Ultra-low temperature freezing	none	Vacuum packaging, modified atmosphere packaging, or freezer-safe containers	Maintain temperature, avoid exposure to air and moisture	2–3 years for most seafood	[57]
5	Freeze-dried	Freeze drying	None or minimal preservatives	Airtight packaging, often in the form of pouches or jars	Room temperature or below, avoid exposure to air and moisture	1–2 years for most seafood	[57]

Packaging is an essential aspect of low-temperature preservation as it helps to protect the quality, safety, and nutritional value of food products during storage and transportation. One of the packaging interventions that can improve low-temperature preservation is barrier packaging. This method involves using materials that prevent the passage of oxygen, water vapor, and other gases that can deteriorate food quality. Additionally, vacuum packaging can be used to remove air from the packaging before sealing it, slowing down the rate of food deterioration. Modified Atmosphere Packaging (MAP) is another intervention that can alter the composition of gases within the packaging to extend the shelf life of food. This process can inhibit bacterial growth and delay the ripening of fruits and vegetables. Lastly, smart packaging can detect changes in temperature and humidity and adjust the atmosphere within the packaging to maintain food quality. In conclusion, packaging interventions such as barrier packaging, vacuum packaging, MAP, and smart packaging can significantly improve the quality, safety, and shelf life of food products during low-temperature preservation [21].

Low-temperature technologies in the seafood industry, crucial for maintaining the cold chain, have traditionally been evaluated using conventional methods that measure sensory, chemical, and physical attributes. However, these methods have limitations, including lack of precision, destructiveness, and complexity. In response, non-destructive evaluation (NDE) technologies have gained prominence in cold chain logistics due to their real-time tracking capabilities and improvements in seafood quality and safety. Recent innovations in NDE technologies within the seafood industry include the integration of machine learning (ML) and artificial intelligence (AI). This integration has given rise to automated detection and classification systems capable of accurately identifying defects and contaminants in seafood products. Such advancements enhance the efficiency of the detection process, reducing the risk of product spoilage and elevating the overall quality and safety of seafood products. Portable NDE devices represent another innovation in the seafood industry, allowing for real-time monitoring of seafood products during transportation and storage. This capability provides seafood companies with greater control over the quality and safety of their products, enabling them to promptly address any issues as they arise [52]. These technological developments contribute to the optimization of cold chain logistics and ensure the preservation of seafood products at optimal quality throughout their journey.

## 6 High temperature technologies for spoilage prevention

**Fig. 6:**
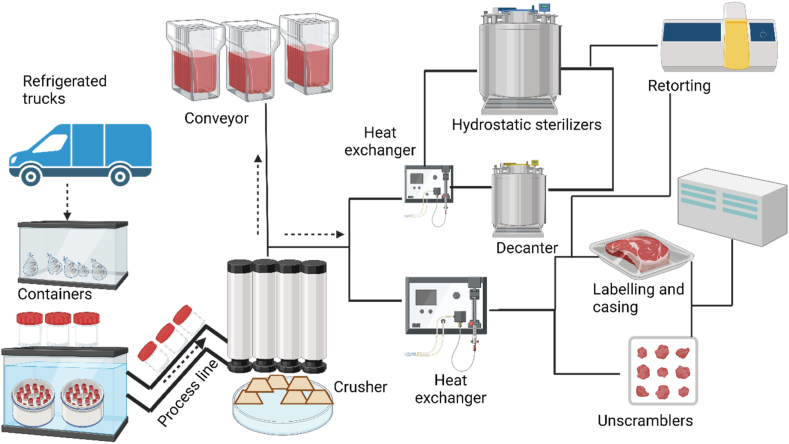
Basic thermal processing concept along with core components (Created with Biorender.com).

Thermal processing is an essential technique used in seafood preservation to maintain the safety and quality of seafood products (Fig. 6). The method involves applying heat to seafood products to destroy harmful microorganisms, enzymes, and other contaminants that can cause spoilage and foodborne illness. The spoilage microorganism in thermally processed seafoods grows due to inadequate appropriate cooling and pre-processing treatment or leaker infection. Thermal processing needs to be applied to inhibit the spoilage and pathogenic bacterial growth and prolong the seafood shelf life. Thermal processing treatment such as deep-frying, roasting, steaming, and boiling, has been applied long time ago. Those thermal processing methods have considerable challenges due to the negative effects on the nutritional quality and sensory properties of the processed products.

To maintain the unintended sensory properties and nutritional quality, various common thermal processing methods such as pasteurization, sterilization, blanching, cooking, smoking, and canning are used. Pasteurization is a common thermal processing method used in seafood processing, where the product is heated to a specific temperature (usually between 60 and 90 °C) for a certain duration to reduce microbial load and extend shelf life [58]. Blanching is used to inactivate enzymes and reduce microbial load before further processing. Cooking seafood products involves heating them to a specific temperature for a certain duration to cook them and eliminate microorganisms. Smoking seafood products involves exposing them to smoke from burning wood or other materials, which imparts flavour and acts as a preservative. Finally, canning seafood products involves heating them to a high temperature (121.1O^O^C) and sealing them in airtight cans to create a sterile environment and extend shelf life. Following proper processing parameters and handling practices is crucial to ensure that processed seafood products are safe for consumption. The core temperature, which is the temperature at the centre of the product, is a critical factor in thermal processing, as it determines the extent of microbial destruction [59]. Fig. 5 shows the basic thermal processing concept, where the products pass through the processing lines, and the heat exchanger is applied to transfer the heat to the sterilization, decanter, packaging steps, or the hot liquid filler (unscramblers). However, the safety of the thermal processing method, such as sterilization, needs to be evaluated based the microbiological risk change of the targeted bacteria surviving during the thermal treatment and based on the lethality achieved. Furthermore, the thermal validation technology needs to be used to validate thermal processing equipment and ensure that processing parameters are within the required limits. Therefore, the heat processing is the most important steps to ensure the product safety during the canned seafood products manufacturing.

In last decades, preservation of seafood using high temperature methods have been reported in Table 4. Besides preservation and sterilization are commonly used, souse vide treatment, ultra-high temperature and microwave-assisted induction heating are recently applied to inhibit the spoilage in seafood [60,61]. Sous vide is another thermal processing technique used in seafood processing, which involves cooking vacuum-sealed seafood products at low temperatures for a longer time to preserve their texture, flavour, and nutritional properties. This method has been applied in various seafood products, such as rainbow trout [62] and European seabass [63], with temperature 50 °C and 90 °C, respectively. The applied different temperatures are used due to the different characteristic of both seafood products, but both methods are effectively prolonging the seafood products, reaching more than a month at refrigerated temperature storage. The disadvantage of this method is that the method needs be combined with other heating processing methods and addition of additives to have more prolonged shelf life of food products. Bongorina [60] investigated the combination between sous vide method and chill for the processed mussels with addition of salt brine as additives, which are able to prolong the shelf life for 30 days at low temperature storage. Other methods combined with sous vide are the pasteurization [64], high-pressure processing [61], and vacuum packaging [63]. The vacuum packaging technology plays a critical role in thermal processing, as it helps to prevent recontamination of the product after processing and additives such as antioxidants, preservatives, and flavour enhancers are sometimes used to enhance the sensory and nutritional properties of seafood products [65]. Furthermore, Ultra-high temperature short time (UHTST) processing is a method of sterilization, where the product is heated to a high temperature for a short duration (usually less than 1 s) to eliminate all microorganisms and extend shelf life [59]. The UHT method was applied for seasoned laver Pyropia spp (Korean seafood), with temperatures reaching 300 °C [66]. This UHT method has more prolonged shelf life, compared to the other heating methods, reaching >12 months.

**Table 4:** High temperature preservation technologies for seafood.

No.	Preservation temperature (Target core temperature)	Type of technology	Packaging material used	Storage conditions	Additives used	Preferred shelf life	Reference
1	50–60 °C	Sous vide	Vacuum-sealed bags	Refrigerated	No additives	2–4 weeks	[59]
2	60–85 °C	Pasteurization	Cans, pouches	Ambient	No additives	2–3 years	[67]
3	90–100 °C	Retorting	Cans or glass jars	Ambient temperature	Salt, acids, and spices	2–5 years	[67]
4	100–121 °C	Canning	Cans or glass jars	Ambient temperature	Salt or spices	2–5 years	[67]
5	>121 °C	High temperature short-time (HTST) Pasteurization	Vacuum sealed bags or MAP	Refrigerated	No additives	2–3 weeks	[67]
6	90 °C or 70 °C	Microwave-assisted induction heating (MAIH)	sealed CPET container	23 °C for 1 h	No additives	∼10 days	[68]
7	300 °C	Ultra-high temperature (UHT)	Not reported	using an air velocity of 20 L/h at 120 °C	No additives	>12 months	[66]
8	Mw: 58.8 ± 2.2 °C Ps: 57.6 ± 1.4 °C	Microwave (Mw) and Microwave pasteurization (Ps)	Vacuum 20-μm polyamide (PA) /70-μm polyethylene bag	24 days refrigerated storage	No additives	24 days	[69]
9	70 °C	Pasteurization and sous vide packaging	Vacuum-packaged in a gas barrier plastic pouch	4 ± 1 °C	Lemon juice	2 weeks	[64]
10	High pressure processing (HPP) and sous vide (SV)	HPP: 150 MPa or 350 MPa SV: 65 °C	NR	28 days of refrigerated storage	No additives	28 days	[61]
11	90 °C	vacuum packed sous-vide	polyethylene polyamide pouch	refrigerated (3 ± 1 °C) conditions for 60 days	laurel (Laurus nobilis) and curcuma (curcuma longa)	45–55 days	[63]
12	85 °C	sous vide cook and chill method	Oriented Polyamide/Polypropylene (OPA/PP) pouch	chilled storage (3.0 ± 1 °C) for 21 days	salt brine	30 days	[60]
13	50 °C and 55 °C	sous-vide	NR	+4 °C for 36 days	antimicrobial essential oil (Rosemary, Coriander, Basil, and Laurel)	36 days	[62]

Note: NR: Not Reported.

Microwave-assisted heating method becomes interesting in seafood preservation due to its intended characteristics, e.g., low maintenance, safe handling, ease of use, and high heating rates. The method work by converting the electromagnetic energy into thermal energy, that can be applied as traditional heating methods, like thawing, extraction, cooking, and sterilization. The benefits of this methods are less sensory and nutritional quality effects in seafood products. Tsai et al. [68], shows the preservation of Barramundi fish at 70 and 90 °C sealed by crystallized polyethylene terephthalate (CPET) container. Besides prolonging the seafood shelf life for 10 days, the method maintain the desirable texture (hardness and chewiness) and optimal colour and appearance of the products. Lerfall et al. [69], evaluated the shelf life and quality of Atlantic salmon fillets treated with microwave and microwave pasteurization, with temperature 57.6–58.8 °C. The method affected the protein denaturation, sensory properties, colour and microbial growth, but did not affect the liquid loss of the salmon packaged with soluble gas stabilization treatment before pasteurization. Furthermore, the method still effectively prolong the shelf life of the seafood for 24 days. However, the application of this method for seafood products are still in consideration due the cost of the equipment hindering wider deployment, especially at large-scaled equipment.

In recent years, besides microwave method, other heating methods becoming an industrial interest and promising alternatives are spectroscopic techniques, infrared heating, radio frequency heating, and ohmic heating. These novel methods are regarded as volumetric heating (internal heating), which are different with conventional heating using convection and conduction process (external heating). The spectroscopic techniques have become increasingly popular for monitoring thermal treatments in fish and other seafood due to their non-destructive and rapid nature. In recent years, there have been several developments and applications of spectroscopic techniques in this field. Raman spectroscopy has been used to detect changes in protein conformation and lipid oxidation during thermal treatments in fish and seafood. Recent studies have shown that Raman spectroscopy can also be used to detect adulteration in seafood products. For instance, it can differentiate between fresh and frozen-thawed fish fillets by identifying changes in their protein and lipid profiles. The infrared heating method includes Near-Infrared Spectroscopy (NIRS), Infrared spectroscopy (IR), and Fourier Transform Infrared (FTIR). NIRS is non-destructive technique that has been used to monitor thermal treatments in fish and other seafood. NIRS can be used to measure changes in the composition of fish muscle during cooking and has been shown to accurately predict the moisture and fat content of cooked fish. Infrared spectroscopy (IR) has also been used for monitoring thermal treatments in fish and seafood. IR can detect changes in protein and lipid structures, as well as identify oxidation products. A recent study demonstrated the use of Fourier Transform Infrared (FTIR) spectroscopy to monitor the oxidative changes in fish oil during thermal treatments. Additionally, UV–visible spectroscopy has been used to monitor thermal treatments in fish and seafood. This technique can detect changes in the colour and pigments of fish during cooking, as well as the formation of Maillard reaction products. Overall, spectroscopic techniques have shown great potential for monitoring thermal treatments in fish and other seafood. With ongoing advancements in technology, these techniques will likely continue to play an important role in the seafood industry for quality control and food safety purposes [[70], [71], [72]].

Another method recently applied for extraction, fermentation, pasteurization, dehydration, evaporation, blanching, thawing, cooking, and heating is ohmic heating. This method has more uniform heating and higher heating rates than the conventional methods. Other benefits are high energy efficiency and environmentally friendly. Ohmic heating was used for Alaska pollock, and pacific surimi gels combined with carrot cuts, resulting effects on the texture of the products [73]. Ohmic heating can also be combined with other non-thermal processing, such as high pressure [74]. In general, the ohmic heating can decrease the total, thawing loss of frozen tuna fish cubes with short thawing time [75].

The seafood industry has undergone significant transformations in recent years, driven by the advent of Industry 4.0, which has facilitated advancements in preservation and processing methods to address emerging thermal challenges like ohmic and microwave heating. Integration of digital technologies, including the Internet of Things (IoT), artificial intelligence (AI), and big data analytics, has brought about notable improvements in seafood processing, preservation, and analytical techniques. In seafood processing, Industry 4.0 technologies such as automation and robotics have played a pivotal role. Automation and robotics optimize production processes, reduce labor costs, and enhance efficiency. Robots, for instance, are utilized for tasks like filleting, portioning, and packaging of fish, reducing manual labor, ensuring consistent quality, and mitigating the risk of contamination. Concerning seafood preservation, Industry 4.0 technologies have spurred the development of innovative techniques. IoT sensors, for example, monitor temperature, humidity, and other conditions during transportation and storage of seafood products, ensuring optimal conditions and reducing the risk of spoilage, thereby improving shelf life. Analytical techniques in the seafood industry have also evolved with Industry 4.0. Big data analytics processes vast amounts of data from sources like processing plants, supply chains, and consumer feedback, leading to enhanced decision-making and improved quality control. AI contributes to predictive analytics, identifying potential issues before they become significant problems. In addition to technological advancements, the seafood industry has seen a heightened focus on sustainability and traceability. The utilization of blockchain technology enables end-to-end traceability of seafood products, from their source to the consumer. This not only ensures sustainability but also enhances transparency and accountability within the industry [76].

## 7 Non-thermal technologies for spoilage prevention

**Fig. 7:**
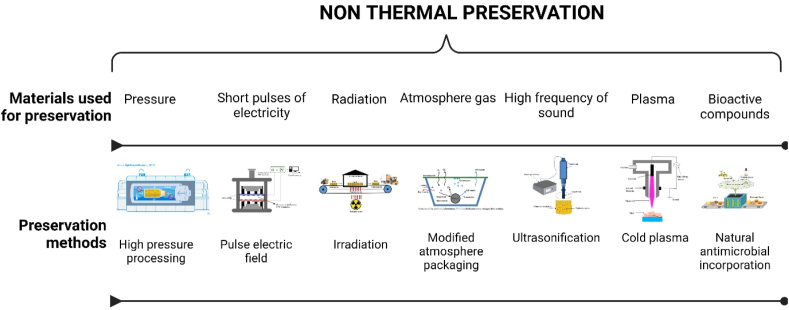
Types of non-thermal processing technologies (Created with Biorender. com).

6. Application of non-thermal technologies has been extensively studied in last decades as increasing consumer demands for minimally processed food product with maintaining nutritional and sensory characteristics. Non-thermal processing technologies (NTPT) (Fig. 7) is an innovative technique used to preserve seafood without using heat. This process involves using different technologies to destroy microorganisms, extend shelf life, and maintain the quality of seafood products. Non-thermal technologies are also associated with minimal heat processing (MHP), which is a promising technology for the seafood industry to extend the shelf life of fish while maintaining its nutritional and sensory quality. Recent advances in MHP of fish have focused on the effects of this technology on microbiological activity and safety. MHP and NTPT technologies such as high hydrostatic pressure (HHP) or High Pressure Processing (HPP), pulsed electric field (PEF), ultrasonication, irradiation, ultrasound technology, pulsed light, packaging technology, addition of bioactive compounds (additives, natural antimicrobials, and bacteriophages) and cold plasma have been investigated for their ability to inactivate microorganisms in fish, as shown in Fig. 6 and Table 5.

**Table 5:** Non-thermal preservation technologies for seafood.

No	Type of technology	Preservation conditions	Packaging material used	Storage conditions	Additives used	Preferred shelf-life	Reference
1	Ultrasonication	20 kHz	Vacuum sealed bags	2–4 °C	None	3–5 days	[77]
2	Cold Plasma (Non-thermal) Technology	Seafood exposed to plasma	Vacuum sealed bags	−18 °C or lower	Natural compounds (chitosan)	6–12 months	[20]
3	Vacuum Packaging	Vacuum-sealed bag	Polyethylene (PE) and polypropylene (PP)	0–5 °C	No additives used	14 days shelf life	[21]
4	Irradiation	Ionizing radiation	No specific packaging material required	0–5 °C	No additives are used	3 weeks	[48,43]
5	Modified Atmosphere Packaging (MAP)	Controlled atmosphere containing a specific mixture of gases such as carbon and nitrogen	polyethylene (PE), polypropylene (PP), and polyethylene terephthalate (PET)	0–5 °C	Antimicrobial agents such as organic acids and essential oils are sometimes added	14 days	[19,21]
6	High Pressure Processing (HPP)	100–1000 MPa for several minutes	Polypropylene or polyethylene terephthalate (PET)	0–5 °C	No additives used	14 days	[78]
7	Pulsed Light Technology	surface exposed to short pulses of high-intensity light	Vacuum-sealed bags or MAP	2–4 °C	None	3–5 days	[79]
8	Ultraviolet (UV) Radiation	surface exposed to UV radiation	Vacuum-sealed bags or MAP	2–4 °C	None	3–5 days	[79]
9	Ionizing Radiation (IOR)	exposed to ionizing radiation	Vacuum-sealed bags or MAP	−18 °C or lower	None	Up to 1 year	[79]
10	Ozone Treatment	Seafood exposed to ozone gas in water or air	Vacuum-sealed bags or MAPs	2–4 °C	None	5–7 days	[80]
11	High-pressure processing (HPP) shucking	300, 400, and 500 MPa for 3 min	Vacuum bag	4 °C	Soy sauce	extended the storage life by 9–15 days	[81]
12	Pulsed electric fields (PEF), carbon dioxide (CO2: 70%), and high hydrostatic pressure (HPP)	PEF: 1–2 kV/cm, 20 μs, Carbon dioxide (CO2: 70%), and HPP: 150 MPa, 5 min	Vacuum-sealed high-density polyethylene (HDPE) bags	4 °C	No additives	7–10 days	[82]
13	Modelling-assisted minimal heat processing	70 °C and 90 °C for 249.5 s, and 930 or 31.5 s, respectively	Vacuum-packed polyethylene/polyamide bag	4–8 °C	No additives	4 and 12 days	[83]
14	High pressure processing (HPP) and mild heating	250 and 350 MPa for 10 min and 75 °C for 5 min, respectively	Vacuum packaged in LDPE films (thickness, 80 ± 0.5 μm)	4 °C	No additives	>25 days compared to 10 days in raw samples	[84]

Note that the actual preservation conditions, packaging materials, storage conditions, and preferred shelf-life may vary depending on the specific type of seafood being preserved and other factors.

HPP is one of the most common non-thermal processing methods applying pressure of up to 600 MPa to seafood products. HPP treatment has been shown to reduce the number of microorganisms in fish, including spoilage bacteria and pathogens. This process can destroy bacteria, viruses, and parasites without affecting the nutritional and sensory properties of the seafood. The method is generally applied to inhibit the microorganisms to extend the seafood shelf life with pressure shift freezing or thawing in addition to deboning procedure of crustaceans and bivalves. The product characteristics, pressure holding time or temperature, and pressure level should be considered in microbial inactivation. The quality of products can be maintained while bacterial inhibition in the pressure levels ranging between 200 and 600 MPa for 2–10 min for then holding times. Therefore, variety of seafood products, oyster, lobster, mackerel, and rainbow trout and etc has been applied using the HPP. HPP can also be combined with other methods such PEF [82], mild heating [84], etc. Combination of the technology shows a longer shelf life of seafood products.

PEF treatment can also reduce bacterial populations to extend the shelf life of seafood products and maintain acceptable sensory and high nutritional quality, although its effectiveness may depend on the characteristics of the fish being processed. PEF method work employing short-duration pulses (1–100 s) in strong electric fields (0.3–4 kV/cm) [85]. However, the Preservation with PEF to seafood products have more oxidation and protein carbonyls than untreated seafood products, as the PEF cause a cell membrane damage during electroporation process [86]. Consequently, the sensory and texture quality changes of seafood products can be positive due to greater tenderization and can be negative because of the reduced nutritional quality and safety and decreased functional properties. Although the PEF is efficient and eco-friendly, the application of PEF in seafood industry is still limited [87]. Pérez-Won et al. [82], investigated the role of PEF, combined carbon dioxide and HPP treatments for chilled coho salmon packaged with vacuum-sealed density polyethylene (HDPE) bags. The treated salmon products stored at refrigerated temperatures have prolonged shelf life till 7–10 days. The result also shown the texture changes in pre and post-rigor, where the hardness and chewiness of the products were improved for the pre-rigor salmon. This study also shows a significant decrease of lipase and protease activities of the pre-rigor salmon fish. Therefore, PEF is potential methods with novel system in preservation of seafood products, that can also be applied in other seafood products.

Another non-thermal processing method is ultrasonication, which uses high-frequency sound waves to destroy microorganisms and improve the texture of seafood. Ultrasonication or ultrasound is known as an emerging and novel non-thermal preservation and processing technology, according to a sound wave with 20 kHz frequency, which is higher than hearing ability of human. The frequency value of ultrasound are divided in low and high energy groups. The low frequency ranges 5-1- MHz with intensity of <1 W/cm^2^ and the high frequency ranges from 20 to 100 kHz with intensity of >1 W/cm^2^ [76]. Both low and high frequency have different functions. The high energy groups are used for disruption and producing chemical and physical alterations in food quality to inhibit the microbial growth and maintaining the seafood products quality, while the low energy groups are applied for food quality non-destructive examination. The ultrasonication produces shear disturbance and cavitation bubbles, create microstreaming, and shear forces, and form free radicals, which caused microbial cell wall disruption and then led to decay and shelf-life extension.

Irradiation is a non-thermal processing method that uses ionizing radiation to destroy microorganisms, parasites, and pests in seafood. Food irradiation is another novel method using radioactive isotopes cobalt (_27_Co^60^) and caesium (_55_Cs^137^) to emit ionizing irradiation, interacting with components of the irradiated foods to generate the biological, chemical, and physical impacts [88,89]. Wei et al. [90], preserved seafood products using electron beam irradiation, that does not need ionizing irradiation from radioactive isotopes electrons. E-beam irradiation was also investigated by Yu et al. [91], to preserve the nutrition and quality of the Atlantic salmon. The study shows an effective inactivation on SARS-cov-2 in refrigerated temperatures, inducing lipid oxidation, decreasing vitamin A, and increasing amino acids. Furthermore, the irradiation caused alteration on texture and chewiness. This quality degradation can be controlled by irradiation dose, in which the advised dose should be 2–7 kGy, but the increasing dose cause more quality degradation. In another study, Damdam et al. [92], investigated the UV-C irradiation, combined with vacuum sealing on the salmon fillets shelf life, in which these combination effectively reduced the growth of bacteria, including *E. coli, Salmonella*, and aerobic bacteria, lactic acid bacteria (LAB) to prolong the shelf life of the salmon by 66.6%. This method shows a good antibacterial effect, but the limitation of the methods in seafood product application can be considered due to potential lipid and protein oxidation, and sensory changes. This limitation can be overcome by combination with antioxidants, that can help in preserving seafood and prolong their shelf life.

Cold plasma treatment has been shown to have antimicrobial effects on both surface and internal bacteria in fish. The cold plasma produced various reactive species, like ultraviolet, negative, and positive ions, free radicals including the reactive nitrogen species (RNS) and reactive oxygen species (ROS). Liao et al. [93], investigated water ice activated using cold plasma to preserve fresh shrimps. The plasma-activated water has significant advantage for the fresh shrimps preservation, due to the capability in bacterial inhibition and prolonging their shelf life for 4–8 days by delaying the deterioration changes in hardness and colour. Although this method is good in improving the organoleptic and nutritional properties of the seafood products, the method has limitation, including oxidation of lipid and protein, discoloration, and alterations in organoleptic properties [94].

Packaging technology plays a crucial role in non-thermal food preservation by protecting the food from contamination and prolonging its shelf life. One of the most common packaging technologies used in non-thermal preservation is vacuum packaging. This technique removes oxygen from the package, creating an anaerobic environment that inhibits the growth of spoilage microorganisms. Another technique is modified atmosphere packaging (MAP), which replaces the air in the package with a controlled gas mixture, such as carbon dioxide and nitrogen. This method can also help to slow down the growth of microorganisms and the rate of spoilage [95].

Overall, non-thermal processing methods offer an effective way to preserve seafood without altering its taste, texture, and nutritional value, making them a valuable tool in the seafood industry [96]. However, they also have some cons, such as the need for specialized equipment, the potential ineffectiveness against some bacterial strains and spores, and the relatively short shelf-life extension compared to other methods. Overall, non-thermal food processing and preservation methods offer a promising alternative to traditional thermal processing methods, especially for raw or minimally processed foods [20,95].

In terms of safety, MHP (Moderate Heat Processing) and NTPT (Non-Thermal Preservation Technologies) have demonstrated their effectiveness in preserving the nutritional and sensory quality of fish while reducing the risk of foodborne illness. However, ongoing research is needed to determine their efficacy against specific pathogens and identify optimal processing conditions. Recent advances in fish processing have shown promise in enhancing microbiological safety while maintaining product quality. The utilization of advanced technologies, natural antimicrobials, and bacteriophages in MHP and NTPT for fish can contribute to reducing the risk of foodborne illness and extending the shelf life of fish products [97]. Natural antimicrobials, such as essential oils, plant extracts, and organic acids, have been explored for their ability to inhibit the growth of microorganisms in fish. Bacteriophages, which are viruses that infect and kill bacteria, have also been investigated for their potential use in controlling bacterial pathogens in fish [98]. Additives like organic acids, enzymes, and bacteriophages can be employed in non-thermal preservation to prevent spoilage. For instance, organic acids such as acetic and citric acids can lower the pH of food, creating an inhospitable environment for microorganisms. Enzymes like lysozyme can break down bacterial cell walls, leading to their destruction. Despite the effectiveness of non-thermal preservation, spoilage can still occur due to factors like air, moisture, or light. Proper packaging materials and techniques, along with appropriate storage conditions, are essential to prevent spoilage. Additionally, maintaining proper sanitation and hygiene practices during processing, handling, and storage is crucial to minimize the risk of contamination. Therefore, a combination of packaging technology, the use of additives, and adherence to proper storage conditions plays a pivotal role in non-thermal food preservation, preventing spoilage, and extending the shelf life of food products (83]. Non-thermal preservation methods offer a promising alternative to traditional thermal processing methods by preserving the nutritional value, flavor, and texture of food. NTPT, such as ultrasound, high-pressure processing, and gamma irradiation, combined with alternative chemical compounds like peracetic acid, bacteriocins, nanoparticles, and essential oils, have proven efficient in inactivating microorganisms, reducing spoilage, and mitigating the impact of environmental conditions on meat and fish products. However, further research and improvements in application conditions are necessary to decrease contamination levels in all types of seafood [99]. Table 6 discusses shelf-life of different seafood preservation technologies in relation to different variables.

**Table 6:** Shelf-life of different seafood preservation technologies in relation to different conditions.

Sl. No.	Preservation technology	Treatment condition	Core Temperature (^o^C)	Storage Temperature (^o^C)	Type of seafood	Shelf life	Reference
1	Chilling	0–4 °C	0–4	0–4	Tilapia (Oreochromis mossambicus)	15 days	[100]
					Atlantic mackerel (*Scomber scombrus*)	7 days	[101]
					shrimp (Penaeus chinensis)	6 days	[102]
					Indian octopus (*Cistopus indicus*)	8 days	[103]
2	Superchilling	−20 to −30 °C	−1	−1 to −4	Nile tilapia (Oreochromis niloticus)	20 days	[104]
					Atlantic mackerel (*Scomber scombrus*)	14 days	[101]
					swimming crab, *Portunus trituberculatus*,	10–15 days	[105]
3	Freezing	−35 to −45 °C	−20	−20	Nile tilapia (Oreochromis niloticus)	29 months	[106]
					Atlantic salmon (Salmo Salar)	360 days	[107]
					lobster (*Homarus americanus*)	12 months	[108]
					Squid (Todarodes pacificus)	365 days	[109]
4	Thermal sterilization	121.1 °C	121.1	10 to 15	Rohu fish (*Labeo rohita*)	12 months	[110]
					Atlantic salmon (Salmo Salar)	>12 months	[111]
5	High Pressure Processing (HPP)	300–700 Mpa/(5 °C – 20 °C)/2–5 min	Na.	0–4	tilapia (*Oreochromis niloticus*)	1 week	[112]
					black tiger shrimp (*P. monodon*)	20 days	[113]
					Blue crab (*Callinectes sapidus*)	18 days	(121)
6	Modified Atmosphere Packaging (MAP)	Gas Ratio (O2/CO2/N2)	Na.	0–4	tilapia (*Oreochromis niloticus*)	>12 days	[114]
					Atlantic Salmon (*Salmo salar*)	22 days	[115]
					Dolphin fish (Coryphaena hippurus)	28 days	[116]
7	Pulsed Electric Field (PEF)	10–80 kV/cm @ micro to milliseconds	Na.	0–4	tilapia (*Oreochromis niloticus*)	8 days	[117]
					Pacific white shrimp (*L. vannamei*)	10 days	[127]

## 8 Practical application of innovative preservation technologies in seafood industry

**Fig. 8:**
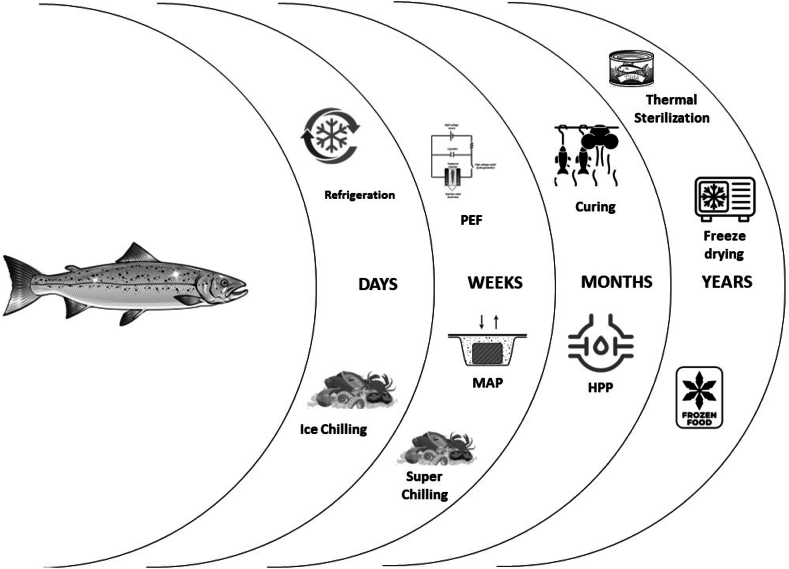
Shelf-life extension potential of different seafood preservation technologies.

Preservation plays a pivotal role in conserving the nutritional quality of seafood for an extended period of time so that the producers are rewarded with best price for their commodity and the consumers with a quality product befitting the price they pay. The increasing demand for sustainable and minimally processed food from the consumer side has resulted in research and development in seafood processing technology focussed on more and more energy efficient and nutritionally non-altering methods [118]. The existing popular preservation methods like chilling, freezing and thermal sterilization is also getting their share of updating inline with the trend. Chilling has been a popular and economical method of fresh seafood preservation since its invention. Low infrastructural, packaging and transportation cost for ice-chilled fresh produce has been revolutional for the industry in extending the shelf-life of seafood for short duration without effecting the consumer acceptability [23]. Modern cold-chain facilities with proper monitoring and smart notification techniques has given much needed boost for the chilled retail seafood market even in places away from coastline or water resources. Rapid chilling of the fresh seafood immediately after their capture is key to the future preservation and shelf life. Super chilling is introduced to extend the shelf life of fresh seafood from days to weeks compared to normal chilled storage [119]. It involves only partial freezing thus not imposing the higher costs involved with actual freezing. Super chilling is really beneficial in the case on bord handling of multiday fishing trips or transporting fresh seafood to distant places without compromising on the quality as well as not enduring quality alterations of deep freezing. Deep freezing is without dispute the most adapted preservation techniques with inherent advantages such as long shelf life, minimal organoleptic and nutritional degradation [120]. The major disadvantage of deep freezing is the cost involved in procurement, instalment, operational manpower, energy costs and logistics expenses attached to it. Irrespective of that, different freezers and technologies provide the producer with options suitable to their product and scale with maximum unit price to their product due the quality imparted by the technology to the produce. Recent developments such as Magnetic field-assisted freezing, Electric field-assisted freezing and High-pressure freezing has resulted in maximising the efficiency of the technology with minimal impact on energy consumption [120]. Freeze drying is another process suitable for preserving moisture sensitive food products such as seafood protein concentrates, hydrolysates, and extracts without losing their colour, texture, or nutritional properties with prolonged shelf life at ambient temperatures [121]. This technique removes moisture through sublimation and have added advantages such a s reduced product bulk, rehydration capacity with concentration of flavours resulting in enhanced taste experience for consumers. The technology is suitable for products with premium pricing category as it requires higher initial investment costs. Thermal sterilization provides consumer products in ready-to-eat form with added advantage of ambient temperature storage and prolonged shelf life [122]. It is a versatile technology suitable for preserving even complex cuisines up to 1 year in room temperature, giving various product options for consumers and producers. The concentrated form of preserved food and recyclability of the packaging gives it a sustainable advantage against other methods. Even though the time temperature combinations could be precisely standardised based on the food variety, it possess certain disadvantages such as high initial investment cost, severe processing conditions resulting in nutritional loss and anaerobic lethal microbial risk if poorly monitored [123]. Various advanced research and development is happening in thermal processing such as Ohmic heating, Microwave assisted thermal sterilization, Radio frequency heating, Infrared heating in order to minimize the heat induced nutritional and sensory degradation and energy consumption without compromising on microbial sterility [124]. Also, more and more precise thermal validation methods using advanced measurement probes are also being introduced for precise calculation of the optimum thermal process for the designated seafood variety. Modern innovative non-thermal methods such as High-pressure processing (HPP), Modified Atmosphere Packaging (MAP), application of natural antimicrobial agents, Pulsed Electric Field (PEF) and Ultrasound technology focusses on extension of shelf life of seafood products with minimal processing and impact on nutritional and sensory characteristics [80]. Even though with higher additional costs, these technologies could be applied in combination with traditional chilled preservation as hurdles with maximising the shelf life of the seafood products. Fig. 8 depicts the shelf-life extension potential of different seafood preservation technologies.

## 9 Conclusions

This assessment of cutting-edge interventions in preservation and packaging technologies for reducing seafood spoilage emphasizes several key findings. Initially, it is evident that seafood spoilage is a multifaceted process influenced by various biological, chemical, and physical factors. Consequently, employing a combination of preservation and packaging methods is often essential for optimal outcomes. Secondly, employing low-temperature technologies like ice, chilling, super chilling, freezing, and freeze-drying proves effective in averting spoilage. Various freezers and freezing media can be utilized based on the specific seafood product. Thirdly, thermal processing emerges as a viable means of preserving seafood, with techniques such as pasteurization, sous vide, UHTST, and sterilization offering diverse preservation levels. Non-thermal methods like high-pressure processing, modified atmosphere packaging (MAP), natural antimicrobial agents, pulsed electric field, and ultrasound also demonstrate effectiveness in spoilage reduction. Addressing the challenge of seafood product variability remains crucial, making it challenging to apply universal preservation techniques. Balancing preservation requirements with sensory and nutritional quality poses another obstacle. Although strides have been made in novel packaging materials, such as biodegradable or active packaging, there's a continued need for cost-effective, readily available materials with minimal environmental impact. Lastly, a call for more sustainability-focused research is emphasized. In summary, while advancements have been achieved in seafood preservation and packaging, there's ongoing necessity for improvements in tailored techniques, sensory and nutritional preservation, packaging materials development, and comprehensive sustainability approaches.

## Funding

The open access publishing fee is covered under the agreement by the DEAL Consortium upon acceptance due to Shahida Anusha Siddiqui being affiliated to the Technical University of Munich.

## Data availability statement

Sharing research data helps other researchers evaluate your findings, build on your work and to increase trust in your article. We encourage all our authors to make as much of their data publicly available as reasonably possible. Please note that your response to the following questions regarding the public data availability and the reasons for potentially not making data available will be available alongside your article upon publication.

## CRediT authorship contribution statement

**Shahida Anusha Siddiqui:** Methodology, Validation, Formal Analysis, Investigation, Resources, Writing – original draft, Writing – review & editing, Visualization, Data Curation, Software, Project administration, Funding acquisition, Supervision. **Shubhra Singh:** Data Curation, Writing – original draft, Formal Analysis, Visualization. **Nur Alim Bahmid:** Data Curation, Writing – original draft, Formal Analysis, Visualization. **Abhilash Sasidharan:** Conceptualization, Data Curation, Writing – original draft, Writing – review & editing, Supervision.

## Declaration of competing interest

The authors declare that they have no known competing financial interests or personal relationships that could have appeared to influence the work reported in this paper.
